# The efficacy of thymosin α1 as immunomodulatory treatment for sepsis: a systematic review of randomized controlled trials

**DOI:** 10.1186/s12879-016-1823-5

**Published:** 2016-09-15

**Authors:** Fang Liu, Hong-Mei Wang, Tiansheng Wang, Ya-Mei Zhang, Xi Zhu

**Affiliations:** 1 Department of Pharmacy, Peking University Third Hospital, Beijing, 100191 China; 2 Department of Pharmacy, Yanqing Teaching Hospital of Capital Medical University/Yanqing County Hospital, Beijing, 102100 China; 3 Department of Clinical Pharmacy and Pharmacy Administration, School of Pharmaceutical Sciences, Peking University, Beijing, 100191 China; 4 Department of Critical Care Medicine, Peking University Third Hospital, 49 North Garden Road, Haidian District, Beijing, 100191 China

**Keywords:** Thymosin α1, Sepsis, Systematic review, Immunomodulatory

## Abstract

**Background:**

Thymosin α1 (Tα1) as immunomodulatory treatment is supposed to be beneficial for the sepsis patients by regulating T cell subsets and inflammatory mediators. However, limited by the small sample size and the poor study design, the persuasive power of the single clinical studies is weak. This meta-analysis aimed to investigate the impact of Tα1 on the sepsis patients.

**Methods:**

We searched for the Cochrane Central Register of Controlled Trials, MEDLINE, EMBASE, CBM, VIP, CNKI, WANFANG, Igaku Chuo Zasshi (ICHUSHI) and Korean literature databases reporting the effects of Tα1 on outcomes in sepsis patients.

**Results:**

Among 444 related articles, 19 randomized controlled trials (RCTs) met our inclusion criteria. Mortality events were reported in 10 RCTs included 530 patients, and the meta-analysis showed significant decrease in Tα1 group compared with control group (RR 0.59, 95 % CI 0.45 to 0.77, *p* = 0.0001). The subgroup analysis showed no difference between the two dosages (RR 0.59, 95 % CI 0.43 to 0.81; RR 0.59, 95 % CI 0.35 to 0.98, respectively). In 9 RCTs, with a total of 489 patients, Tα1 administered once per day decrease APACHE II score significantly (SMD −0.80, 95 % CI −1.14 to −0.47, *p* < 0.0001) while Tα1 twice per day showed no effect (SMD 0.30, 95 % CI-0.10 to 0.70, *p* = 0.14). However, the length of ICU stay, the incidence of multiple organ failure (MOF) and duration of mechanical ventilation were not significantly affected by Tα1 treatment (SMD −0.52, 95 % CI −1.06 to 0.11, *p* = 0.06; SMD −0.49, 95 % CI −1.09 to 0.11, *p* = 0.11; SMD −0.37, 95 % CI −0.90 to 0.17, *p* = 0.17, respectively). As to the immunological indicators, the level of HLA-DR were increased by Tα1 (SMD 1.23, 95 % CI 0.28 to 2.18, *p* = 0.01) according to the pooled analysis of 8 studies involving 721 patients. Lymphocyte subsets CD3, CD4 and cytokines IL-6, IL-10 and TNF-α were also beneficially affected by Tα1 treatment.

**Conclusions:**

Tα1 may be beneficial to sepsis patients in reducing mortality and modulating inflammation reactions. However, the quality of evidence supporting the effectiveness is low considering the small sample sizes and inadequate adherence to standardized reporting guidelines for RCTs among the included studies.

## Background

Sepsis is a major cause of morbidity and mortality in developed countries [1]. The mechanism of the sepsis syndrome is not completely understood though we do know it includes a systematic immune system response in multiple and complex pathways which is named SIRS (Systemic Inflammatory Reaction Syndrome) [2, 3]. Sepsis begins with epitope shifting from antigen presenting cells into neutrophils, macrophages and T helper lymphocytes (Th), followed by cell transcription factor NF-k B activating, entering nucleus and forming a complex with DNA. Subsequently, apoptosis is induced and Th lymphocytes is activated into Thl cells, which release a large amount of proinflammatory cytokines and chemokines, such as TNF-α, IL-6, IL-1β, IFN-γ and monocyte chemoattractant protein (MCP-1), then complement and coagulation system were activated, and systematic inflammation was developed, leading to high fever, shock, coagulation dysfunction and multiple organ failure, and even death [4–6].

Thymosin α1 (Tα1) is an acidic polypeptide consisting of 28 amino acids extracted and purified from the thymosin fraction 5. Pharmacological studies showed that Tα1 stimulates endogenous IFN-γ secretion, and enhances T cells and the whole immune system [7–12]. Pharmacokinetic studies in healthy volunteers showed good absorption after subcutaneous injection with a peak serum level at between 1 and 2 h and a half-life of less than three hours [13]. Tα1 is approved mainly in countries of Asia and South America, for the treatment of chronic hepatitis B and C as a vaccine enhancer [14]. Although some clinical trials demonstrated that Tα1 is beneficial for the treatment of sepsis by regulating T cell subsets and inflammatory mediators [15–17], the results are less persuasive due to the small sample size and the poor study design. As the influence of Tα1 on prognosis of patients with sepsis remains inconclusive, this systematic review aims to quantitatively evaluate the efficacy and safety of Tα1 in the treatment of sepsis.

## Methods

### Inclusion criteria

Studies are included if the following criteria are met: 1) Randomized controlled trials (RCTs); 2) Evaluating adult sepsis patients. We defined sepsis according to internationally accepted diagnostic criteria developed on 2001 SCCM/ESICM/ACCP/ATS/SIS international sepsis definitions conference [18]. 3) Comparing Tα1 as add on therapy with no treatment or placebo on the basis of standard or conventional treatment of sepsis in both groups. Standard or conventional treatment is defined as regular treatment for sepsis including adequate empiric antibiotic therapy, ventilation regimen, blood glucose control, resuscitation and hemodynamic support, organ support, sedation or analgesia as needed and adequate nutrition.

### Outcome measures

The primary outcomes are death from any cause assessed 28, 60 and 90 days after the initiation of treatment assignment, the length of ventilation and the length of ICU staying. The secondary outcomes included dynamic changes of Sequential Organ Failure Assessment (SOFA), multiple organ dysfunction syndrome (MODS), Acute Physiology and Chronic Health Evaluation II (APACHE II), T lymphocyte subsets, CD4^+^/CD8^+^, monocyte human leukocyte antigen-DR (mHLA-DR) expression, and cytokines including IL-6, IL-10 and TNF-α measured on day 0 (the day of enrollment) and 7 in both groups. The rate of adverse drug reactions was taken as indicator for tolerability.

### Search strategy

We searched Cochrane Central Register of Controlled Trials (CENTRAL) (The Cochrane Library, Issue 3 of 12, March 2016), MEDLINE (January 1966 to April 19, 2016), EMBASE (January 1980 to April 19, 2016) for published studies and Clinicaltrials for registered studies in English [19]. We searched China National Knowledge Infrastructure (CBM), VIP Database for Chinese Technical Periodicals (VIP), Chinese National Knowledge Infrastructure (CNKI) and Wanfang Data in Chinese, all from inception to April 19, 2016. We searched Igaku Chuo Zasshi (ICHUSHI) for Japanese literature, and Korean literature up to February 12, 2015 [20]. We checked the bibliographies in reports of the randomized trials, review articles, and meta-analyses to identify other potentially eligible studies. We used a combination of keywords related to the names of thymosin α1 (Tα1 or Thymosin-alpha (1) or Thymalfasin or Thymalfasine or Thymalsasinum or Timalfasina or Zadaxin) and the type of sepsis-associated disease (“severe infection” or “sepsis” or “septic shock”).

### Study selection

Two review authors (FL and HMW) checked titles and abstracts identified from the register, obtained the full text of all potentially relevant studies for independent assessment. The authors decided independently which trials fitted the inclusion criteria and resolved disagreements by discussion or consulting the third author (XZ). The reasons for excluding studies from the review were documented and justified.

### Data extraction and management

Two review authors (FL and HMW) performed data extraction independently with a pre-tested electronic table. Discrepancies were resolved by consensus or a third author’s (XZ) adjudication. The following data were abstracted from each study: characteristics of the studies, characteristics of the included patients and outcomes of the studies. The first or corresponding author of each included study was contacted for clarifications and further information when required.

### Assessment of risk of bias in included studies

We used a domain-based evaluation as recommended by the Cochrane Handbook 5.0.2 for Systematic Reviews of Interventions [21]. The following domains were assessed: 1) random sequence generation; 2) allocation concealment; 3) blinding of participants, personnel and outcome assessors; 4) incomplete outcome data; 5) Selective reporting. 6) Bias from other source. We graded these items as having high, low or unclear risk. When discrepancies between review authors existed, we reassessed the studies and reached agreement by consensus.

### Statistical analysis

We calculated the treatment effect across trials using the Cochrane statistical package, Review Manager 5.3 (RevMan). We expressed results as risk ratios (RR) with 95 % confidence intervals (CIs) for dichotomous outcomes, such as mortality, and mean differences (MDs) and 95 % CIs for continuous outcomes, such as the length of ventilation, the scores of the evaluation scales, the counts of lymphocytes subsets and the concentration of cytokines. Heterogeneity among studies was assessed using a Chi^2^ test of heterogeneity (*P* value < 0.1) and the I^2^ statistic [22].

Trials comparing similar regimens were pooled using fixed effect model, unless significant heterogeneity was observed when useing random-effects model. If the mean and SD of the continuous outcomes were not reported in the studies, we assigned the median as the mean if sample size was greater than 25 and estimated the SD from the range (that is, SD range 0.95/4 or interquartile range/1.35) as suggested by Hozo et al. [23]. If sample size was less than 25 we used formulas suggested by Hozo et al. to calculate the mean [23]. If we could not calculate the mean or SD from the available data, we excluded the study from the analysis.

### Sensitivity analysis

We undertook sensitivity analyses taking into account the quality of the studies. To evaluate a single study’s effect on the pooled data, sensitivity analysis was carried out by excluding each study. Publication bias was evaluated using Funnel plots and Fail-Safe Number (Nfs) [24].

### Subgroup analysis and investigation of heterogeneity

We explored sources of heterogeneity with a priori subgroup hypotheses: dosage regimen of Tα1. Patients received subcutaneous injections of 1.6 mg Tα1 (ZADAXIN™, SciClone Pharmaceuticals, Foster City, CA, USA) twice per day for 5 consecutive days, then once per day for 2 consecutive days.

## Results

### Characteristics of included studies

**Fig. 1:**
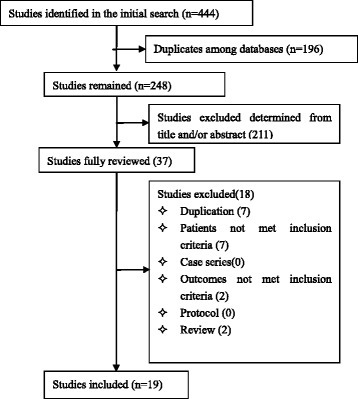
Flow diagram for study selection. A detailed flowchart of the search and selection results is shown in this figure

From electronic searches and hand searches,we retrieved 444 relevant publications. A total of 248 articles were obtained from initial screening, and 19 RCTs involving 1354 adult patients were included in the meta-analysis. A detailed flowchart of the search and selection results is shown in Fig. 1. All the included studies [25–43] were conducted in China. The key characteristics of included trials were summarized in Table 1. The prior or preexisting conditions were addressed as burn, hospital-acquired pulmonary infection and abdominal infection respectively in three studies [28, 29, 37], however, the patients included in these studies comply the diagnosis criteria of sepsis. Of the 19 RCTs, 14 included studies [25–36, 42, 43] used Tα1 1.6 mg per day and 5 studies [37–41] used 1.6 mg, twice per day, both administered subcutaneously.

**Table 1:** Characteristics of included studies. SSC therapy: Surviving Sepisis Campaign therapy

Study	Population	Case number, Tα1/control	Interventions in Tα1 group	Interventions in control group	Outcomes
Chen XL 2009 [25]	Sepsis patients in ICU, age over 18 years	40, 20/20	SSC therapy + Tα1, 1.6 mg,SC,QD	SSC therapy + NS	Levels of CD3,CD4,CD8, CD4/CD8, NK,CRP, APACHE II
Cheng AB 2010 [26]	Sepsis patients in ICU, age under 70 years and HLA-DR < 30 %	60,30/30	Conventional treatment + Tα1, 1.6 mg, SC, QD	Conventional treatment + NS	Levels of CD4,CD8 and HLA-DR
Gui CM 2012 [27]	Sepsis patients in ICU, age between 18 and 80 years	42,22/20	SSC therapy + Tα1, 1.6 mg,SC,QD	SSC therapy	Levels of CD4, CD4/CD8, igg, iga, igm, PCT, IL-6, IL-10 and APACHE II
Hu XL 2007 [28]	Abdominal sepsis patients in ICU	45,24/21	Conventional treatment + Tα1, 1.6 mg, SC, QD	Conventional treatment + NS	Levels of TNF-α, IL-6, IL-10, CD3,CD4,CD8, CD4/CD8, NK and 28-day mortality
Gong ZH 2011 [29]	Burn sepsis patients	56,28/28	Conventional treatment + Tα1, 1.6 mg, SC, QD	Conventional treatment	Levels of TNF-α, and WBC
Chen J 2007 [38]	Septic shock, APACHE II scores between 15 and 20	42,21/21	SSC therapy + Tα1, 1.6 mg,SC, BID	SSC therapy	Levels of T-lymphocyte subtype, natural killer cell and mechanical ventilation time, length of ICU stay, 28-day mortality
Fan JB 2014 [30]	Sepsis patients or septic shock	120,60/60	Conventional treatment + Tα1, 1.6 mg, SC, QD	Conventional treatment	Levels of CD4, CD8, CD4/CD8, APACHE II and 28-day mortality
Lei S 2005 [37]	Severe hospital acquired pneumonia patients in ICU, HLA-DR <30 %,	38,21/17	Conventional treatment + Tα1, 1.6 mg, SC, BID	Conventional treatment	Levels of CD4, CD8, CD4/CD8, NK, HLA-DR and 28-day mortality
Li YN 2009 [31]	Age over 18 years, suffering from severe sepsis with Marshall score over 5	47, 23/24	SSC therapy + Tα1, 1.6 mg,SC, QD	SSC therapy	Levels of HLA-DR, CD3, CD4, CD8, length of ICU stay, APACHE II, 28-day mortality and mechanical ventilation time
Wu JN 2004 [32]	Sepsis patients in ICU, HLA-DR <30 %	44,22/22	Conventional treatment + Tα1, 1.6 mg, SC, QD	Conventional treatment + NS	Levels of HLA-DR, CRP, APACHE II; and MOF
Wu JF 2013 [39]	Patients in ICU with severe sepsis	361,181/180	Conventional treatment + Tα1, 1.6 mg, SC, twice per day for 5 consecutive days, then once per day for 2 consecutive days	Conventional treatment + NS	Levels of HLA-DR, CD4/CD8, WBC, duration of ICU stay, mechanical ventilation time, APACHE II and 28-day mortality
Wu JF 2014 [40]	Sepsis patients, age over 18 years	54,26/28	Conventional treatment + Tα1, 1.6 mg, SC, twice per day for 5 consecutive days, then once per day for 2 consecutive days	Conventional treatment	28-day mortality
Zhang BJ 2014 [41]	Sepsis patients	60,30/30	Conventional treatment + Tα1, 1.6 mg, SC, twice per day	Conventional treatment	Level of IL-6 and APACHE II
Zhang Z 2006 [33]	Sepsis patients	38,19/19	Conventional treatment + Tα1, 1.6 mg, SC, QD	Conventional treatment	Levels of CRP, CD3, CD4, CD8, CD4/CD8, NK, and APACHE II
Zhou LX 2009 [35]	Severe sepsis aged > 18, Marshall score > 5	47, 23/24	Tα1 plus SSC therapy	SSC therapy	IL-6, IL-10, TNF-α, HLA-DR, T lymphocytes, 28-day mortality
Zhao MY 2007 [34]	Sepsis patients in ICU, HLA-DR <30 %, age <70	42,21/21	Conventional treatment + Tα1, 1.6 mg, SC,QD	Conventional treatment + NS	Levels of HLA-DR, CD4, CD8, TNF-α,IL-6 and IL-10
Zhou Q 2011 [36]	Severe sepsis, age > 18 years	82,42/40	SSC therapy + Tα1, 1.6 mg,SC,QD	SSC therapy	Levels of HLA-DR, CD3, CD4, CD8, CD4/CD8
Zhu 2015 [43]	Severe sepsis, age > 18 years	60,30/30	Conventional treatment + Tα1, 1.6 mg, SC, QD	Conventional treatment + NS	Levels of, CD3, CD4, CD8, CD4/CD8, duration of ICU stay and APACHE II
Lu 2015 [42]	Patients with siai, age > 18 years	76,38/38	Conventional treatment + Tα1, 1.6 mg, SC, twice a week	Conventional treatment + NS	Levels of, CD3, CD4, CD8, CD4/CD8

### Risk of bias in included studies

The quality of the included studies was assessed using the criteria defined in the Cochrane Handbook 5.0.2 [21]. The assessments and grades given are shown in Table 2. Only 2 out of 19 trials were considered as high quality.

**Table 2:** Risk of bias in include studies

The first author	Publication year	Adequate sequence generation	Allocation concealment	Blinding (Participant)	Blinding (Personnel)	Blinding (Outcome assessor)	Incomplete outcome data (attrition bias)	Selective reporting (reporting bias)	Other bias
Chen XL	2009	Unclear	Unclear	Unclear	Unclear	Unclear	Low risk	Low risk	Low risk
Cheng AB	2010	Unclear	Unclear	Unclear	Unclear	Unclear	Unclear	Low risk	Low risk
Gui CM	2012	High risk	High risk	Unclear	Unclear	Unclear	Low risk	Low risk	Low risk
Hu XY	2007	Unclear	Unclear	Unclear	Unclear	Unclear	Unclear	Low risk	Low risk
Gong ZH	2011	Low risk	Unclear	Unclear	Unclear	Unclear	Low risk	Low risk	Low risk
Chen J	2007	Unclear	Unclear	Unclear	Unclear	Unclear	Low risk	Low risk	Low risk
Fan JB	2014	Unclear	Unclear	Unclear	Unclear	Unclear	Unclear	Low risk	Low risk
Lei S	2005	Unclear	Unclear	Unclear	Unclear	Unclear	Unclear	Low risk	Low risk
Li YN	2009	Unclear	Unclear	Unclear	Unclear	Unclear	Low risk	Low risk	Low risk
Wu JN	2004	Low risk	Low risk	Unclear	Unclear	Unclear	Unclear	Low risk	Low risk
Wu JF	2013	Low risk	Low risk	Low risk	Low risk	Low risk	Low risk	Low risk	Low risk
Wu JF	2014	Low risk	Low risk	Low risk	Low risk	Low risk	Low risk	Low risk	Low risk
Zhang BJ	2014	Unclear	Unclear	Unclear	Unclear	Unclear	Low risk	Low risk	Low risk
Zhang Z	2006	Unclear	Unclear	Unclear	Unclear	Unclear	Unclear	Low risk	Low risk
Zhou LX	2009	Unclear	Unclear	Unclear	Unclear	Unclear	Low risk	Low risk	Low risk
Zhao MY	2007	Low risk	Low risk	Unclear	Unclear	Unclear	Low risk	Low risk	Low risk
Zhou Q	2011	Low risk	Low risk	Unclear	Unclear	Unclear	Low risk	Low risk	Low risk
Zhu SJ	2015	Unclear	Unclear	Unclear	Unclear	Unclear	Low risk	Low risk	Low risk
Lu FP	2015	Unclear	Unclear	Unclear	Unclear	Unclear	Low risk	Low risk	Low risk

### The impact on mortality

None of the included studies reported 60 and 90-day mortality.

### Primary analysis of 28-day mortality

**Fig. 2:**
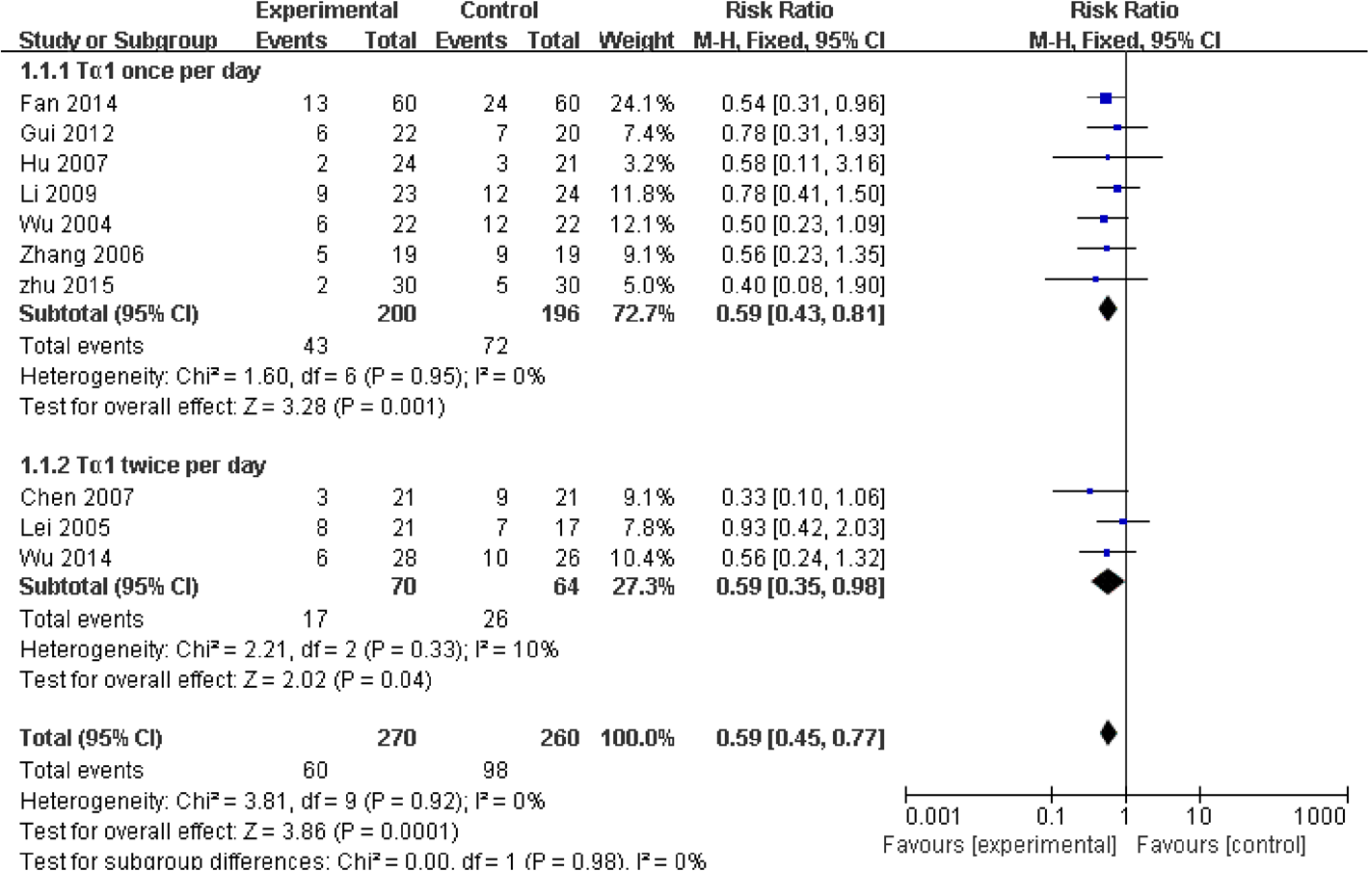
The effect of thymosin α1 on 28-day mortality. A total of 10 studies reported mortality within 28 days, and it included a total of 530 patients and 158 events

**Fig. 3:**
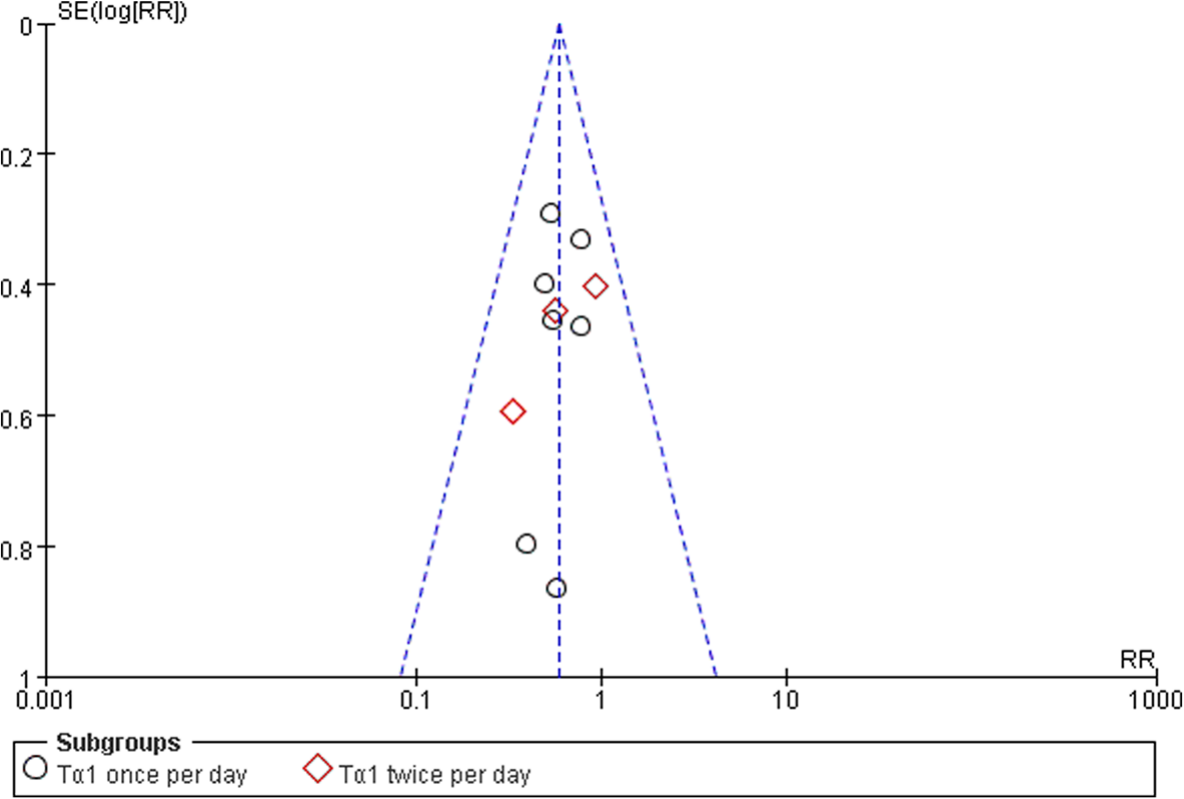
Funnel plot of the published studies in relation to the 28-day mortality meta-analysis. Ten studies were included. No evidence of a publication bias in a funnel plot analysis

A total of 10 studies reported mortality within 28 days, including a total of 530 patients and 158 events (Fig. 2). No significant heterogeneity was found across the 10 studies (Chi 3.81, I^2^ 0 %, *p* = 0.92). Furthermore, we detected no evidence of publication bias after a funnel plot analysis (Fig. 3), and Nfs_0.05_ = 36.04. The RR showed a significant decrease of mortality in Tα1 group compared with control group (RR 0.59, 95 % CI 0.45 to 0.77, *p* = 0.0001) (Fig. 2).

### Subgroup analysis of 28-day mortality

To explore the relationship between different dose of Tα1 and 28-day mortality, we conducted the subgroup analyses. The intervention of Tα1 administered once per day was adopted in 7 trials involving 396 patients and the intervention of Tα1 administered twice per day was adopted in three trials involving 134 patients. The subgroup analysis showed both the two dosage regimens significantly decreased mortality of sepsis patients (Tα1 once per day: RR 0.59, 95 % CI 0.43 to 0.81; Tα1 twice per day: RR 0.59, 95 % CI 0.35 to 0.98) (Fig. 2).

### The impact on APACHE II

#### Primary analysis

**Fig. 4:**
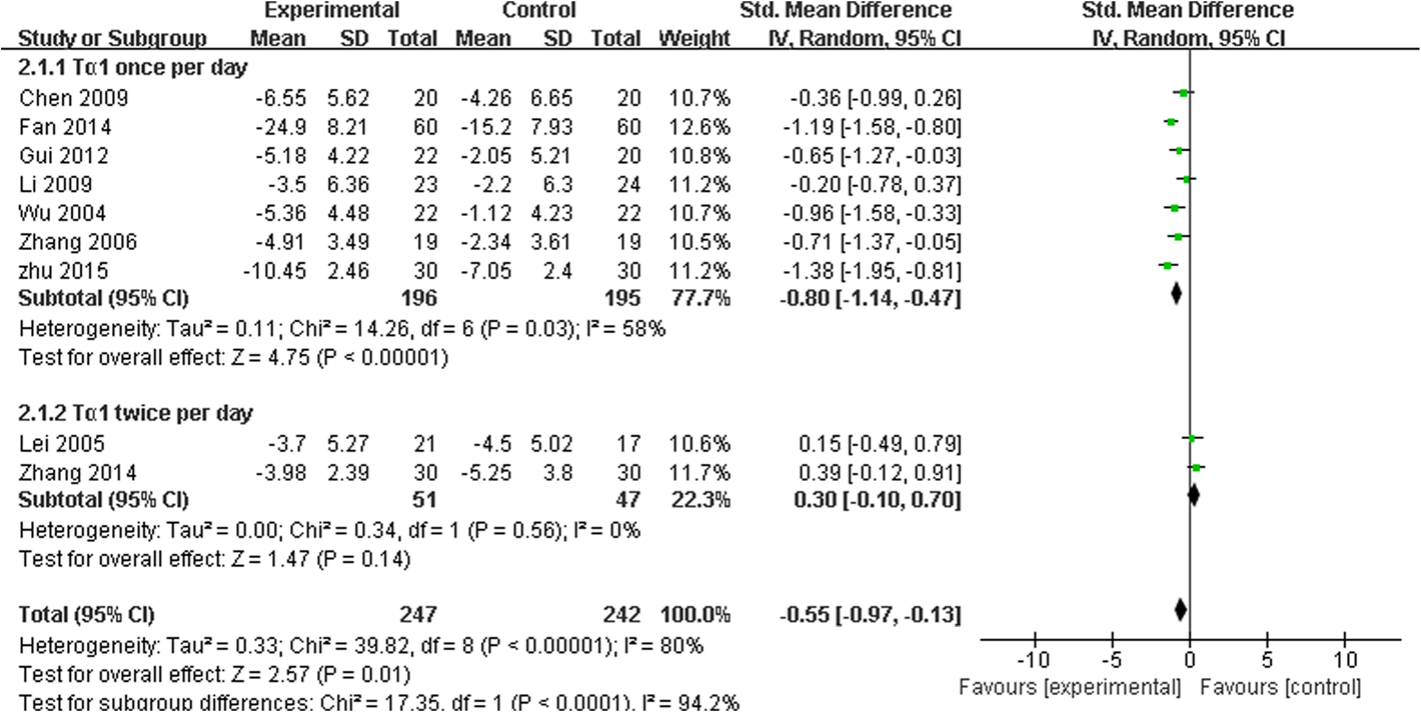
The effect of thymosin α1 on APACHE II. Nine studies reported APACHE II score, and 489 patients were included. There was a significant difference in APACHE II score between thymosin α1 and control group

Nine studies involving 489 patients reported APACHE II score. There was a significant difference in APACHE II score reduction between Tα1 and control group (SMD −0.55, 95 % CI −0.97 to −0.13, *p* = 0.01), which meant that Tα1 decreased APACHE II in a greater degree than control group. Since the heterogeneity was high (Chi 39.82, I^2^ 80 %) (Fig. 4) among different studies, we conducted subgroup analysis.

#### Subgroup analysis

The intervention of Tα1 administered once per day was adopted in 7 trials involving 391 patients and the intervention of Tα1 twice per day was adopted in two trials involving 98 patients (Fig. 4).

SMD for Tα1 once per day group was −0.80 (95 % CI −1.14 to −0.47, *p* < 0.00001) with a moderate heterogeneity (Chi 14.26, I^2^ 58 %, *p* = 0.03).

However, the effect of Tα1 twice per day on APACHE II was not statistically significant (SMD 0.30, 95 % CI-0.10 to 0.70, *p* = 0.14). No significant heterogeneity was found across the 2 studies (Chi 0.34, I^2^ 0 %, *p* = 0.56).

#### The impact on MOF

Only one study involving 44 patients reported the incidence of multiple organ failure (MOF). As shown in Table 3, there was no significant difference on MOF between Tα1 and control group. (SMD −0.49, 95 % CI −1.09 to 0.11, *p* = 0.11).

**Table 3:** The influence on MOF, length of ICU stay and mechanical ventilation days

	Included studies	Cases	Chi	I^2^ %	SMD	95 % CI	*P*
MOF	1 [32]	44	–	–	−0.49	−1.09,0.11	0.11
Length of ICU stay	6 [31, 33, 35, 37, 39, 43]	591	34.92	86 %	−0.52	−1.06,0.01	0.06
Mechanical ventilation days	6 [31, 33, 35, 37–39]	570	32.24	84 %	−0.37	−0.90, 0.17	0.17

#### The impact on mechanical ventilation days

Six studies reported duration of mechanical ventilation, and a total of 570 patients were included. As shown in Table 3, there was no significant difference on the mechanical ventilation days between Tα1 and control group (SMD −0.37, 95 % CI −0.90 to 0.17, *p* = 0.17). However, heterogeneity was high (Chi 32.24, I^2^ 84 %, *p* < 0.001).

#### The impact on length of ICU stay

Six studies involving 591 patients reported the length of ICU stay. As shown in Table 3, there was no significant difference on the length of ICU stay between Tα1 and control group. SMD was −0.52 (95 % CI −1.06 to 0.01, *p* = 0.06) with high heterogeneity (Chi 34.92, I^2^ 86 %, *p* <0.0001).

#### The impact on HLA-DR levels

Eight studies including 721 patients reported the level of HLA-DR. There was a significant difference in HLA-DR between Tα1 and control group. SMD was 1.23 (95 % CI 0.28 to 2.18, *p* = 0.01), with a high heterogeneity (Chi 179.65, I^2^ 96 %) (Fig. 4). To explore the high heterogeneity among different studies, we conducted subgroup analysis.

#### Subgroup analysis

**Fig. 5:**
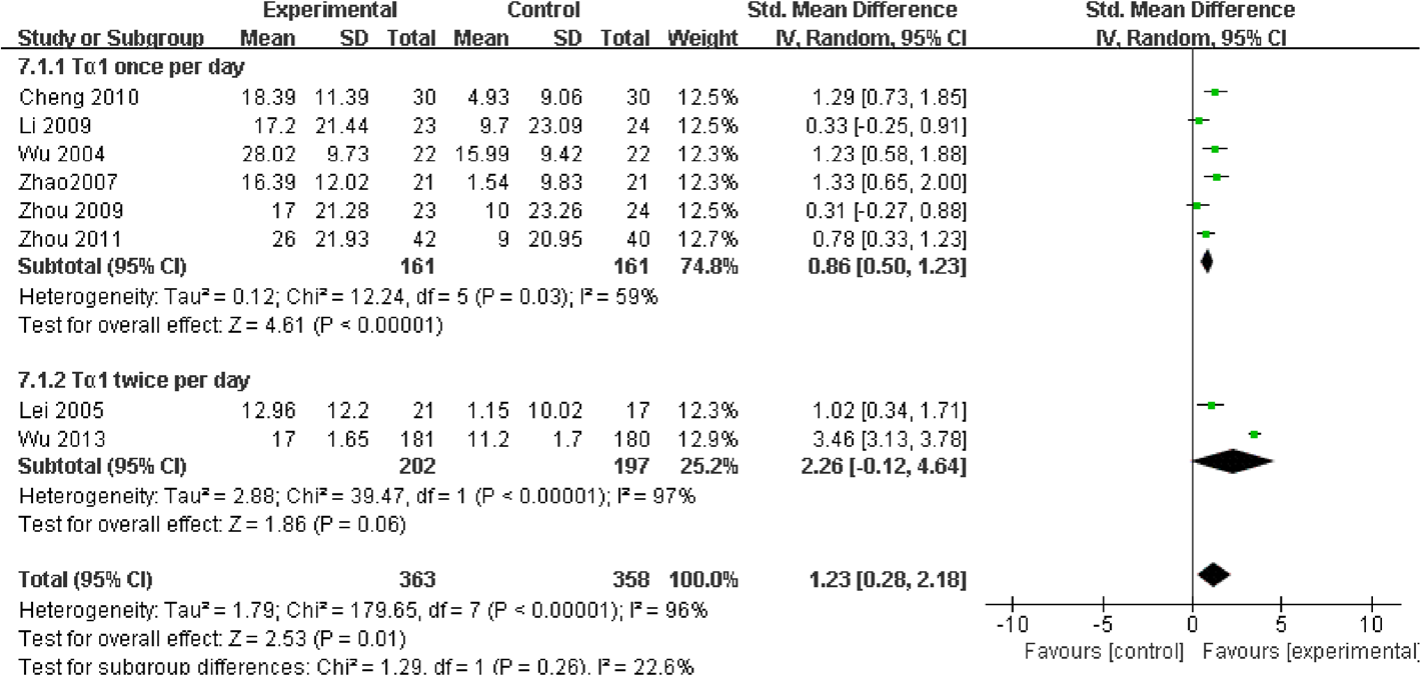
The effect of thymosin α1 on HLA-DR levels. Eight studies including 721 patients reported the level of HLA-DR. There was a significant difference in HLA-DR between thymosin α1 and control group

The group of Tα1 administered once per day included six trials with 322 patients and the group of Tα1 administered twice per day included two trials with 399 patients. The subgroup analysis showed a significant effect of Tα1 once per day on HLA-DR (SMD 0.86, 95 % CI 0.50 to 1.23, *p* < 0.001) with a moderate heterogeneity (Chi 12.24, I^2^ 59 %, *p* = 0.03). However, the effect of Tα1 twice per day on HLA-DR was not statistically significant (SMD 2.26, 95 % CI-0.12 to 4.64, *p* = 0.06) with a significant heterogeneity (Chi 39.47, I^2^ 97 %, *p* < 0.001) (Fig. 5).

#### The impact on T lymphocyte subsets

Tα1 showed significantly better effect on CD3^+^, CD4^+^ and CD4^+^/CD8^+^ than control, but didn’t show difference on CD8^+^. The pooled results were showed in Table 4. However, there was a high heterogeneity across these studies.

**Table 4:** The influence on lymphocyte subsets

Lymphocyte subsets	Included studies	Cases	Chi	I^2^ %	SMD	95 % CI	*P*
CD3	9 [25, 28, 31, 33, 35, 36, 38, 42, 43]	521	56.12	86 %	0.84	0.35,1.33	0.0008
CD4	14 [25–28, 30, 31, 33–38, 42, 43]	779	51.01	75 %	0.80	0.50,1.10	<0.0001
CD8	12 [25, 26, 28, 30, 31, 33–37, 42, 43]	695	62.64	82 %	−0.27	−0.64,0.10	0.16
CD4/ CD8	13 [25, 27, 28, 30, 31, 33, 35–39, 42, 43]	1038	102.37	88 %	0.62	0.22,1.02	<0.0001

#### The impact on cytokines

##### IL-6 levels

Four studies involving 189 patients reported the level of IL-6. There was no significant difference on the level of IL-6 between Tα1 and control group (SMD −0.32, 95 % CI −1.24 to 0.60, *p* = 0.49), and the heterogeneity across the four studies was high (Chi 28, I^2^ 89 %, *p* < 0.0001) (Table 5).

**Table 5:** The influence on cytokines (interleukins and TNF-α)

Cytokines	Included studies	Cases	Chi	I^2^ %	SMD	95 % CI	*P*
IL-6	4 [27, 28, 34, 41]	189	28	89 %	−0.32	−1.24,0.6	0.49
IL-10	3 [27, 28, 34]	129	2.63	24 %	1.06	0.64,1.49	<0.00001
TNF-α	4 [28, 29, 34, 35]	190	2.55	0 %	−0.47	−0.76,–0.18	0.002

##### IL-10 levels

Three studies involving 129 patients reported the level of IL-10. There was a significant difference on the level of IL-10 between Tα1 and control group (SMD 1.06, 95 % CI 0.64 to 1.49, *p* < 0.00001). No significant heterogeneity was found across the 3 studies (Chi 2.63, I^2^ 24 %, *p* = 0.27) (Table 5).

##### TNF-α levels

Four studies involving 190 patients reported the level of TNF-α. There was a significant difference on the level of TNF-α between Tα1 and control group (SMD −0.47, 95 % CI −0.76 to −0.18, *p* = 0.002). No significant heterogeneity was found across the 5 studies (Chi 2.55, I^2^ 0 %, *p* = 0.47) (Table 5).

##### Safety of Tα1

The included RCTs reported neither Tα1 related severe adverse event nor treatment discontinuation due to intolerance or adverse events of Tα1.

## Discussion

In this systematic review of RCTs including1354 patients with sepsis, we found benefits of Tα1 on both survival and other clinical indicators. We also explored the efficacy of Tα1 on immune parameters.

### Overall completeness and applicability of evidence

The trials were identified following a systematic search of the literature in multi-language databases. Besides English and Chinese databases, we additionally searched Japanese and Korean database to enhance our systematic review’s ability of reflecting international practice. Study inclusion criteria were tightly defined and the meta-analysis was rigorously conducted according to a predefined analysis plan addressing specific hypotheses.

We didn’t set limitations on the primary etiologies of sepsis, however, all trials included critically ill patients where a common systemic inflammatory pathway was activated. Therefore, we think that there is a good biologic reason to perform a broad meta-analysis, which also considerably increases the generalizability and usefulness of the review.

The data of lymphocyte subsets and cytokines were collected at the 7th day of treatment course of Tα1 in most of the included studies and had the same tendency of favoring Tα1 group as 28 days’ mortality, which indicates that those data can be served as prognosis indicator for sepsis. Our systematic review is consistent with recent studies indicated that the relationship of cytokines and mortality of sepsis [44–46].

In subgroup analysis, both Tα1 1.6 mg once daily and 1.6 mg twice daily decreases mortality, APPACHE II score, ventilation days and ICU days, and they also showed positive effect on lymphocyte subsets and cytokines. Though we didn’t carried out comparisons between the two regimens, we recommend Tα1 1.6 mg once daily to be used for cost-effectiveness considerations.

### The role of Tα1 in immune modulatory therapy of sepsis

It was indicated in a variety of studies that Tα1 modulated immune functions through multiple pathways; however, its mechanism was not fully established [47]. Recent studies suggested that Tα1 combines to toll like receptors (TLRs) located in the surface of dendritic cells (DC), and thus activates them into effector cells with the function of stimulating or inhibiting T cells [48]. As highly specialized type of antigen-presenting cell (APC), DC activate CD3^+^ (total T cells), CD4^+^ (helper T cells), and CD8^+^ (cytotoxic T cells), which is considered as important pathway for Tα1 to reverse immune suppression in sepsis. Moreover, plasmacytoid dendritic cells promote the function of regulatory T Cells, which increase the production of anti-inflammatory factors, such as IL-10 and TGF-β, and reduce the pro-inflammatory cytokines, such as IL-2, IL-6 and TNF-α, so that to combat against the pro-inflammatory cytokines storm in early period of sepsis and then modulate the over-stimulating of nonspecific immunity in the deferment period later on [49]. HLA-DR is expressed in the surface of B-lymphocytes, macrophages, activated T lymphocytes and other immune cells, and the decline of HLA-DR expression is proposed as a reflection of immunosuppression in critically ill patients [50].

Our study shows Tα1 increased CD3^+^, CD4^+^, and CD4^+^/CD8^+^, as well as the level of HLA-DR, which comply with the results of basic research. However, Tα1 did not demonstrate a significant impact on CD8^+^. As to the influence on cytokines, our study showed that Tα1 decreased the level of TNF-α and increased IL-10, but had no significant effect on IL-6. Besides that, it was suggested by both basic research and clinical trials that multiple kinds of other cytokines experience significant changes with the progress of sepsis, including IL-2, IL-3, IL-4 and IFN-γ etc. However, those cytokines were barely evaluated in the included studies. Therefore, more studies, both fundamental and clinical are needed for further understanding the immune-modulatory effect of Tα1 on different immune cells and cytokines with the progress of sepsis.

### Safety of Tα1

According to package insert of Tα1, the rate of adverse reactions of Tα1 is less than 1 % across all its indications. The reported ADRs include pain, redness, and transient muscle atrophy in injection site, multiple joint pains with swelling, and rash. Both Li [51] and our systematic reviews included adverse reactions to evaluate Tα1’s tolerability, and no severe ADRs were recorded in the included clinical trials.

### Comparisons with published review

Three similar systematic reviews had been published recently [51–53]. All of them took Tα1-based immune modulatory therapy as intervention, which means they included not only Tα1, but also concomitantly used ulinastatin. Han [52] evaluated combination of ulinastatin and Tα1, but not Tα1 monotherapy. For Tα1 monotherapy groups, the recently published reviews included fewer studies than ours, which may because they searched less Chinese literature database. For outcomes, all the systematic reviews took mortality as primary outcomes, while durations of mechanical ventilation and ICU stay were evaluated in both Han and Feng’s review, and APACHE II in Feng’s review. Compared to that, our study also included MOF score, hoping to provide some hint on efficacy of Tα1 in organ dysfunction in sepsis though we didn’t come to a definite conclusion because of the limited number of included study. Furthermore, the studies by Li and Feng [51, 53] failed to include immune indicators, while Han evaluated TNF-α and IL-6 [52]. We considered more immune indicators since it was implicated in previous studies shat Tα1 showed pleiotropic effects on immune system [54]. We hope the meta-analysis of immune parameters will be helpful to find out the factors to predict the efficacy of Tα1 in sepsis. Furthermore, providing communitive clinical evidence as meta-analysis may indicate directions for subsequent fundamental researches on the immunomodulatory mechanism of Tα1 in treating sepsis.

We didn’t include studies evaluating efficacy of concurrent use of Tα1 and ulinastatin, except for the studies where ulinastatin were used as background therapy. According to present study, it seems that immune-modulatory effects between Tα1 and ulinastatin overlap each other especially when it comes to their effects on proinflammatory mediators. To figure out to what extent Tα1 is responsible for the beneficial effects noted in the clinical trials, we decided to focus on the studies evaluating the efficacy of Tα1 monotherapy. Future studies providing head-to-head comparison between Tα1 and ulinastatin may be beneficial to further discover the pathway by which immune modulators pose influence on physiopathology of sepsis.

### Limitations of the review

The meta-analysis combined data from a group of predominantly underpowered single center studies. Although there was minimal heterogeneity among trial results on mortality, we are aware that we pooled clinical trials with high risk of bias, thus, the validity of our meta-analysis may be criticized. Another concern is that great heterogeneity existed in the meta-analysis of lymphocyte subsets and cytokines, which may be related to the different measure methods across the included studies. We used standardized mean deviation and carried out sensitivity analysis, and it was showed that the pooled results were stable after removal the studies with heterogeneity.

In 2015, the Society of Critical Care Medicine proposed the new definition of sepsis, which demonstrated sepsis as a life-threatening organ dysfunction (OD) due to a dysregulated host response to infection. SOFA was the major tool to evaluate organ dysfunction, and was shown to be associated with prognosis of sepsis [55]. We included SOFA in our secondary outcomes, regretfully, none of the included studies used SOFA to evaluate efficacy of Tα1.

## Conclusion

In summary, Tα1 may have some benefits in reducing 28 day mortality, deceasing APPACHE II score and modulating immune parameters in sepsis patients, however, the quality of evidence is low. More high-quality studies are needed to confirm Tα1 efficacy in improving clinical outcomes and provide comprehensive understanding of its immumodulatory role in sepsis.

### Key messages

- In sepsis patients, Tα1 decreased 28 days mortality on the basis of regular therapy.
- Both Tα1 1.6 mg once daily and 1.6 mg twice daily had the effect to decrease mortality and APPACHE II score.
- Tα1 increased the level of IL-10 among sepsis patients.
- Tα1 reduced the level of TNF-α among sepsis patients.
- Sepsis patients benefited from Tα1 as immunomodulatory treatment.

## Abbreviations

IL: Interleukin
95 % CIs: 95 % confidence intervals
APACHE II: Acute physiology and chronic health evaluation II
mHLA-DR: Monocyte human leukocyte antigen-DR
MODS: Multiple organ dysfunction syndrome
RCT: Randomized controlled trial
RR: Relative risk
SMD: Standard mean difference
SR: Systematic review
TNF: Tumor necrosis factor
Tα1: Thymosin α1
